# Characterisation and non-pharmacological treatment of Complex Regional Pain Syndrome in under-18-year-olds: a scoping review

**DOI:** 10.1007/s00431-026-07027-w

**Published:** 2026-05-08

**Authors:** Mahira Budhraja, Ryan Purvis, Katrina Tang, Jennifer Lewis

**Affiliations:** 1 School of Health and Social Wellbeing, Centre for Health and Clinical Research, University of the West of England, Glenside Campus, Blackberry Hill, Bristol, BS16 1DD UK; 2 Bath National Pain Centre, Royal United Hospitals NHS Trust, Bath, UK

**Keywords:** Complex Regional Pain Syndrome, CRPS, Paediatric, Pediatric, Paediatric chronic pain, Children and young people, Rehabilitation, Physiotherapy, Occupational therapy, Treatment, Diagnosis, Clinical presentation

## Abstract

The aim of this review was to explore the published literature describing the presentation, characteristics, and non-pharmacological treatments of Complex Regional Pain Syndrome (CRPS) in under 18-year-olds. This scoping review was conducted in line with the Preferred Reporting Guidelines for Scoping Reviews (PRISMA-ScR). Five online databases (AMED, CINAHL + , EMBASE, EBSCO, and MEDLINE) were systematically searched with identified key terms for relevant records and screened against the eligibility criteria. Extracted data from the included studies informed a narrative synthesis. The PAGER framework was used to succinctly present gaps in the field and recommend future directions for research. Online searches yielded 140 articles. Following screening, 30 articles were included for review. Findings suggest primary symptoms included extreme pain, allodynia, and nail changes. The Budapest criteria, radiography, bone scans and laboratory examinations were commonly used diagnostic methods. Various non-pharmacological interventions were used, such as physiotherapy (home exercise), hydrotherapy, desensitisation, and cognitive behavioural therapy.

*Conclusion*: The most common symptom in children is severe pain that is disproportionate to the inciting event. Critical gaps in the evidence base such as the absence of age appropriate, validated diagnostic criteria, lack of research understanding the lived experience and impact of the condition or exploring treatment efficacy, were identified as knowledge gaps within the field.

**Table:** 

**What is Known:** • *Complex Regional Pain Syndrome is a difficult to diagnose and hard to treat pain condition. Among children, it is not well documented or understood, there are no agreed diagnostic criteria or treatment guidelines*.	
**What is New:** • *This scoping review summarises and presents the published research regarding presentation, diagnosis and non-pharmacological treatment of paediatric CRPS*. • *Issues relating to the use of treatments lacking demonstrable efficacy and the need for quick and reliable diagnoses are discussed*. • *Using the PAGER Framework, this review exposes critical gaps in both research (the lack of research around condition experience and treatment efficacy) and practice (the lack of validated diagnostic criteria and treatment pathways) outlining clear and urgent recommendations*.

**Supplementary Information:**

The online version contains supplementary material available at 10.1007/s00431-026-07027-w.

## Introduction

Complex Regional Pain Syndrome (CRPS), a debilitating chronic primary pain condition affecting a limb, is well characterised in adults with agreed diagnostic criteria [1]. The assessment, treatment and management of adult CRPS is established in both UK [2] and international clinical guidelines [3, 4]. However, to date, CRPS in children and young people under 18 years (paediatric CRPS, (pCRPS)) has not been well documented.

Within adult populations, CRPS is typically triggered by trauma to a limb; the condition is characterised by severe pain, disproportionate in magnitude and duration to the initial injury [1]. Accompanying symptoms and signs include colour and temperature changes, swelling and sweating, skin, hair and nail changes alongside movement dysfunction and weakness [1, 2]. The Budapest criteria are the recognised diagnostic method [1] and incidence is 26.2 per 100,000 person years [5].

Among children and adolescents, studies describing symptoms and signs in pCRPS are limited, and the conditions’ natural course is not well understood [6]. The presenting characteristics typical of pCRPS are not yet established. There is debate about whether CRPS in under 18-year-olds is different to that in adults [7] Consequently, there are no agreed diagnostic criteria for under 18-year-olds [7–9]. Without these parameters, CRPS management in this age group is unclear [10]. Given the lack of clinical guidance, access to appropriate treatment and rehabilitation is often convoluted [8] causing distress and negatively impacting patient outcomes [8, 9]. Further, without effective treatment, pCRPS can potentially develop into long-term functional impairment and associated disability, persisting well into adulthood [10]. Uniquely in paediatric populations, chronic pain can affect a child’s development, physical activity, social development, and academic performance [10].

Given these unique considerations, it is essential that we understand the current state of evidence relevant to CRPS in under 18-year-olds. Previous reviews have focused on treatment alone without considering the variation in diagnostic approaches that might affect the specificity of the population being reported [11–13]. Additionally, one review addressing clinical presentations in pCRPS focused only on movement disorders [14].

On this basis, we wish to advance our understanding by answering the question “How is CRPS characterised, diagnosed and treated in under 18-year-olds?”.

Objectives of this review are to:

- Describe the characteristics and presentation of CRPS in children and young people under 18 years old.
- Understand the methods used to diagnose pCRPS.
- Present the range of reported non-pharmacological treatment interventions used to manage pCRPS.

## Methods

A scoping review was conducted to systematically summarise current literature regarding the presentation, diagnosis, and treatment of pCRPS, and identify existing gaps in knowledge. Considering the broad nature of the research question, a scoping review was the most appropriate approach as it allows for a wide range of evidence to be explored in an emerging topic. Registered as an A Priori protocol on Open Science Framework (OSF 10.17605/OSF.IO/8PEMX), this review is reported in line with the Preferred Reporting Items for Systematic Reviews and Meta Analyses extension for scoping reviews (PRISMA-ScR) [15]. See supplementary materials 1 for the complete checklist.

### Search strategy

A comprehensive literature search was conducted in March 2024 for outputs published between 1995 and 2024 by the researchers (RP & KT) on the following databases: AMED, CINAHL +, EMBASE, EBSCO, and MEDLINE. To evidence reproducibility, a blind search of MEDLINE using the search strategy, was conducted by a research librarian at the university and outputs were compared to check for consistency. Databases were searched electronically using keywords and search terms identified using the Participant/Patient, Concept, and Context Framework (PCC) [16]. The framework helped delineate prominent search terms included in the search strategy; the PCC framework also guided the eligibility criteria. The complete search string can be found in supplementary materials 2 (Table 1). Reference lists of relevant articles were checked to collate any articles missed in the database search (February 2025).

**Table 1:** Inclusion and exclusion criteria for studies in this review

Studies were included if they met the following criteria	Studies were excluded if they met the following criteria
Publications between 1995 to date as the clinical definition of CRPS was established in 1995	Publications prior to 1995
Studies with participants aged < 18 years old with Complex Regional Pain Syndrome (CRPS) **or** CRPS studies reporting data and outcomes for children separately to adults	Studies focusing only on adults **or** Studies not reporting separate data for adults and children with CRPS
Studies including: 1. Clinical descriptors for symptoms and signs of pCRPS 2. Diagnostic methods for pCRPS 3. Non-pharmacological interventions for pCRPS	Studies reporting on any other outcomes
Complete journal articles available through university licensed databases or other official channels	Published abstracts **or** Articles unavailable through official channels
Systematic literature review, cohort studies, case reports, observational studies, interviews and qualitative studies	Randomised control trials
Peer reviewed articles published in academic journals	Grey literature, specifically posters, podcasts, websites, and unpublished articles
Articles written in the English language **or** Articles with English translations available	Articles in any language apart from English **or** Articles without English translations available
Schools, hospitals, clinics or any other health and care settings	Any settings not relevant to clinical practice

### Study selection

Articles were selected based on the criteria laid out in Table 1. In line with objective one, randomised control trials (RCTs) were considered outside the scope of this review as based on our knowledge of the field we were seeking articles with detailed breadth and depth of characteristics and symptom presentation. Previous reviews in the field have documented and commented on pharmacological treatment efficacy for pCRPS [11, 12, 17].

### Study screening

**Fig. 1:**
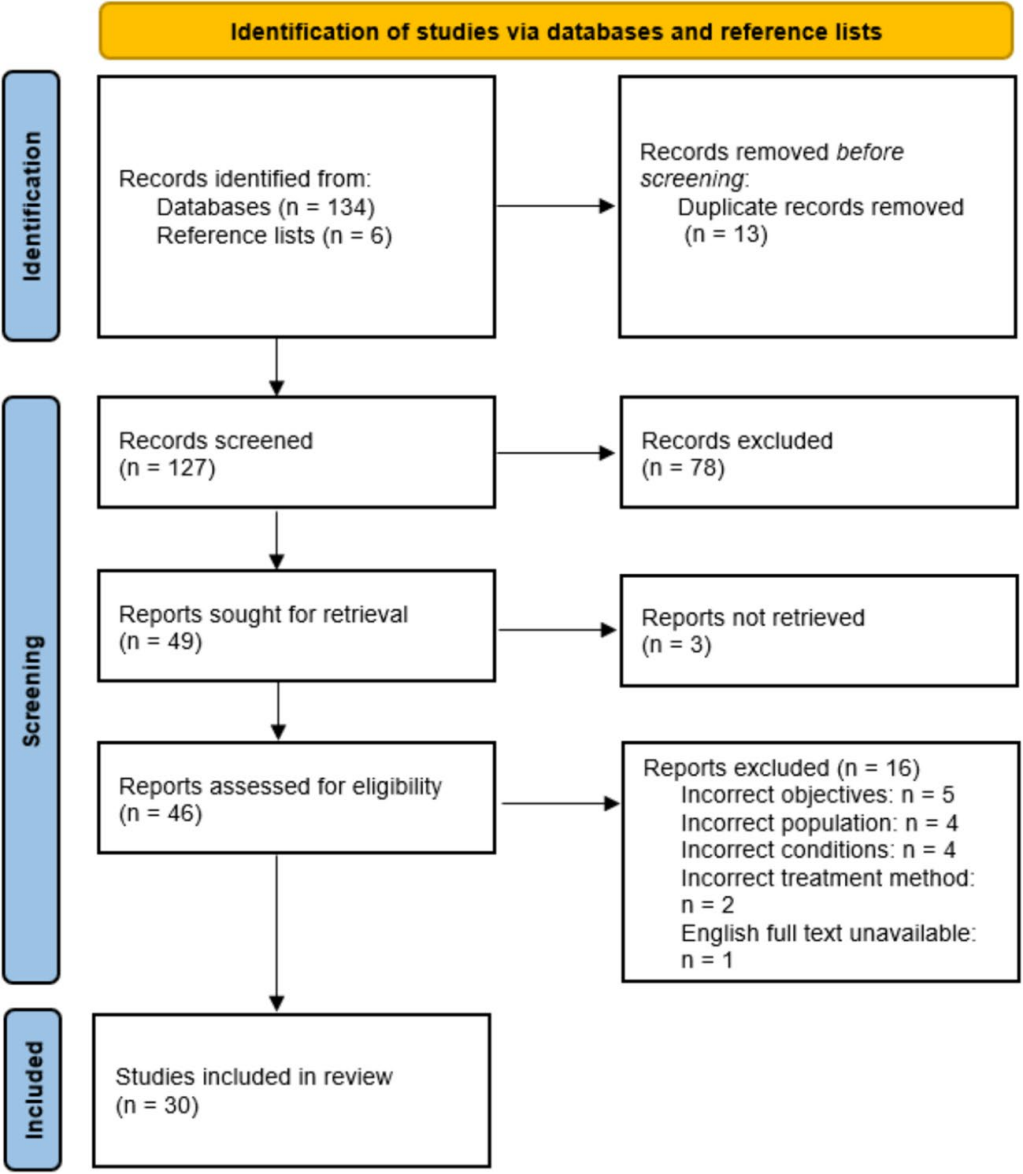
PRISMA-Scr procedure of selecting articles for review

One hundred thirty-four studies were extracted from two online repositories (OVID & EBSCO) where databases AMED, CINAHL +, EMBASE, EBSCO and MEDLINE were accessed, and six studies were found through reference list searches. These were exported to a reference manager (Mendeley, version 1.18.8) and deduplicated prior to title and abstract screening. Title and abstract screening was conducted independently by three reviewers (RP, KT, & KW). Forty-six studies moved to the full text screening where three reviewers reviewed each study against the inclusion criteria. Any discrepancies were resolved through group inspection and discussion. For a detailed breakdown of the study screening procedure see Fig. 1.

### Data charting and synthesis

Data was extracted using an adapted version of the JBI Manual for Evidence Synthesis tool based on the PCC framework [16]. Data was extracted by three reviewers and results were entered into a bespoke Excel form. Any disagreements were resolved through group inspection and discussion. To ensure reliability, MB checked the extracted data against the original manuscripts. Following data extraction, data was grouped across the three primary domains (presentation, diagnosis and treatment) by the first author. This grouping was used to guide the narrative synthesis.

### Quality appraisal

Given the nature of this review, various evidence sources were used to understand the present landscape of literature. Therefore, quality appraisal of the articles was outside the scope of this review [18].

### Implications for research and practice

The PAGER framework for scoping reviews [18] was applied to the synthesised data to enhance the applicability of the findings. The PAGER Framework aims to provide a consistent framework for the reporting and analysis of scoping reviews. Tabulating our results using the PAGER Framework also communicates our findings in a brief and accessible way to reach a variety of audiences. Gaps in knowledge and recommendations for practice were identified and discussed in the context of the results. The framework was used to highlight critical gaps in the literature for pCRPS across presentation, diagnosis and treatment and identify key areas for future research.

## Results

Initial searches yielded 140 articles. After deduplication (*n* = 13), 127 titles and abstracts were screened against the eligibility criteria. Of these, 49 articles moved to full text screening. Following full text assessment of 46 articles, a total of 30 studies were included in this review (see Fig. 1 for details).

### Characteristics of the reviewed studies

Studies were published between 1997 and 2023, with most publications occurring in the last two decades and from the UK or US (15/30). Participants in these studies ranged from age 2 to age 18. From the 30 included studies, 29 reported the presentation of pCRPS, 25 reported the diagnostic methods, and 25 reported the non-pharmacological treatment used. For further details see supplementary files 2, Table 2.

### Presentation

The incidence and prevalence of pCRPS was estimated to be between 1.14 and 1.16/100,000 person years [6, 19]. pCRPS was reported more commonly among girls [6, 10, 14, 19–34] and in the lower limb [6, 10, 13, 14, 19–34]. Precipitating factors included minor trauma (sprains/twists), surgery, fractures, injuries and infections (28/30) [6, 10, 13, 14, 19–42]. Spontaneous occurrences or where a precipitating factor could not be identified were also reported (13/30) [6, 19, 20, 22, 23, 25, 26, 29–32, 34, 43]. Mean age range of pCRPS onset was 11.5–13.3 years [6, 10, 14, 19–21, 24, 26, 41] and pCRPS occurred predominantly in early adolescence, alongside the onset of puberty [32]. One study suggested a younger age of onset for boys in comparison to girls (8.9 vs.12.1 years); however, this article included only two male participants [26].

The primary symptom of pCRPS was pain disproportionate to the trauma experienced [6, 10, 13, 19–21, 23–43]. Pain was described as severe burning, shooting, stabbing, or electrical [10, 21, 35]. Allodynia was reported in 21 articles [6, 10, 19–24, 26–29, 32, 34, 36–40, 42, 43]. Other commonly reported symptoms included skin discoloration (red, purple or blue; 16/30) [6, 10, 19, 22, 23, 25, 26, 28, 29, 31, 36, 39–43], skin temperature changes (particularly coldness; 11/30) [13, 20, 21, 24, 27, 29, 31, 34, 38, 39, 43], oedema (10/30) [6, 13, 19, 22, 23, 26, 27, 31, 35, 43], and decreased range of motion (10/30) [19, 25–27, 31, 37–39, 42, 43]. Less commonly documented symptoms included altered hair (4/30) [10, 21, 27, 28] and nail growth (5/30) [10, 25, 28, 31, 37], hyperalgesia (6/30) [10, 21–23, 27, 40], dystonia (3/30) [6, 21, 24] and muscle atrophy in the affected limb (3/30) [28, 31, 36]. Some articles discussed changes in the nervous system leading to altered sensations (like numbness and tingling) and involuntary movements (tremors, spasms etc.) (3/30) [13, 25, 31]. Two studies recorded functional neurological symptoms, like blindness, stiffness and paralysis [29, 39]. Where reported, participants presented with a wide symptom duration from 0 to 85 months (Average = 15.75 months) [28–30, 33, 36]. For further details see Table 2.

Few studies addressed psychosocial factors associated with pCRPS (8/30) [10, 20, 21, 23, 24, 34, 37, 41]. Anxiety and depression were reported comorbidities in this population (2/30) [21, 34]. Additionally, some studies reported dysfunctional family dynamics (2/30) [20, 23], and sleep disturbances (2/30) [19, 36].

## Diagnosis

The average time from onset to diagnosis was 6 months (range = 0–120 months) [6, 19, 20, 23, 24, 27–29, 33, 36, 41]. Many studies reported physical/clinical examination and history taking as the primary method of diagnosis [10, 13, 21, 22, 24, 32, 35, 37, 38, 40]. Only a third used the Budapest criteria for diagnosing CRPS (10/30) [6, 13, 19, 20, 23, 24, 30, 33, 34, 36]. Investigations were often used to aid diagnoses, commonly reported investigations included radiographs (13/30) [10, 13, 19, 20, 23, 26, 27, 34, 35, 37, 38, 40, 43], bone scans (9/30) [20, 23, 25, 27, 32, 34, 35, 40, 43], and rheumatological tests (7/30) [10, 20, 21, 27, 34, 35, 43]. Imaging studies and MRIs (10/30) were also used (see Table 2) [20, 21, 23, 26, 27, 34, 36, 39, 40, 43]. One study suggested that investigations were primarily conducted to rule out other causes, as a diagnosis was often one of exclusion [13]. Studies that reported the average number of investigations per child, suggested this ranged between 2.3 and 3 investigations prior to diagnosis [23, 27]. Patients were diagnosed by a range of specialties including orthopaedics [26, 39], anaesthesiologists [22, 44], pain physicians [23, 26, 30, 33], and paediatricians [6, 14, 19, 27, 41, 44].

**Table 2:** Presentation and diagnosis of pCRPS

Author/published year	Study design	Sample characteristics	Demographic characteristics	Precipitating factors	Presenting characteristics	Diagnostic method
**Abu-Arafeh & Abu Arafeh, 2016, UK**	Surveillance study	*n* = 26 CRPS type 1 and 2 Age: 5–15 (Mage = 11.9) F/M: 19/7	73% female 73% LL CRPS 54% R side affected 38% multiple site involvement 11.9 yrs M age at onset and diagnosis 2 ms (range 1–12) Md duration onset to diagnosis 1.16/100 000 estimated pCRPS incidence	73% trauma 4% suspected nerve injury 23% spontaneous	Pain, allodynia Oedema Skin discolouration and temperature changes, Loss of function, abnormal movements and dystonia	Budapest Criteria IASP Criteria
**Abu-Arafeh & Abu Arafeh, 2017, UK**	Review	N/A	85% female 76% LL CRPS 15% secondary site 5% on same side 10% opposite side 12.5 yrs M age at onset	71% trauma	N.R	N.R
** Badri et al., 2011, Tunisia **	Case report	*n* = 1 CRPS type 1 Age: 13 F	UL CRPS	Injury	Burning pain Palmar hyperhidrosis Oedema Nocturnal exacerbation Paroxysmal crises post heat exposure	Physical examination *Investigations:* bone scans, radiography and rheumatological tests
** Baerg et al., 2022** **Canada**	Surveillance study	*n* = 168 CRPS (type N.R.) Age: 2–18 (Mage = 12.2) F/M:140/28	83% female 80% LL CRPS 89% single site 12.2 yrs M age at diagnosis 6ms M duration onset to diagnosis 1.14/100,000 estimated minimum pCRPS incidence rising after 12Yrs	65% trauma or injury 7% surgery 20% spontaneous 4% unknown 4% other	Pain, allodynia Skin discolouration Oedema Limited ROM	Budapest Criteria *Investigations:* plain radiograph
**Bayle Iniguez et al., 2015** **France**	Retrospective study	*n* = 73 CRPS type 1 Age: 5–16 (Mage = 11.5) F/M: 64/9	88% female 89% LL CRPS 44% multiple site 25% bilateral symptoms 11.5yrs M age at diagnosis 14.2ms M duration onset to diagnosis (range 0–120 ms)	45% minor trauma 16% spontaneous 4.1% surgery	Pain, allodynia Complete loss of function Coldness and cyanosis Nocturnal exacerbation *Psychosocial factors* 26% family conflict 16% disease or death of relative 11% school issues 3% sexual abuse 1% gender orientation disorder 1% witnessing terrorist attack	Budapest Criteria *Investigations* radiographs, MRI, bone scan, rheumatological tests
** Borucki & Greco, 2015** **USA**	Review	N/A	Mostly females (6 times more likely) 6 to 1 ratio LLCRPS 12–13 yrs M age of onset 12 ms M time to diagnosis but reducing over time, now range 1–41 weeks	5–14% injuries, minor trauma, fractures 10–15% Surgery	Pain (burning, shooting electrical), allodynia, hyperalgesia, Swelling Altered hair growth Motor symptoms and dystonia Coldness, mottling, sudomotor abnormalities *Psychosocial factors:* Anxiety and depression	History and physical examination *Investigations*: Motor strength testing, X-ray, MRI, CT scan, EMG, rheumatological tests
** Broman et al., 2021** **Germany**	Prospective study	*n* = 28 health care professionals	N.R	N.R	N.R	N.R
** Brooke & Janselewitz, 2012** **USA**	Retrospective Study	*n* = 32 CRPS type 1 Age: 8–18 (Mage = 14.3) F/M: 26/6	81% female 44% LL CRPS 31% had neck, back, abdomen, or torso 9% had multiple sites 13.3 yrs M age at onset 9ms M duration between symptom onset and treatment (range = 0.5–48 months)	53% injury or trauma; 44% psychological diagnosis	Pain, hyperesthesia Swelling Skin discolouration and temperature changes	Guidelines for diagnosing pCRPS by Stanton et al. 1993
** Chalkiadis, 2001** **Australia**	Prospective study	*n* = 44 CRPS type 1 and 2 Age: 9–17 (Mage = 13.7) F/M: 33/11	75% females 75% LL CRPS	41% trauma 14% surgery 20% spontaneous	Allodynia, hyperalgesia, Oedema, Skin discolouration and temperature changes Altered sweating	Clinical examination Self or carer reported pain intensity
** di Vadi et al., 2002** **UK**	Case series	*n* = 2 CRPS type 1 Age: 10 and 13 F: 2	UL CRPS	50% minor trauma 50% fracture	Severe constant pain and allodynia Skin temperature changes Limited ROM	N.R
** Dunbar & Wilson, 2019** **New Zealand**	Case report	*n* = 1 CRPS type 1 Age: 15 F	LL CRPS 18 ms pain duration	Minor trauma	Severe pain and allodynia Skin discolouration Swelling Numbness and tingling Poor sleep Muscle atrophy Difficulty weightbearing	Budapest Criteria *Investigations*: MRI
** Güler-Uysal et al., 2003** **Turkey**	Case report	*n* = 1 CRPS type 1 Age: 2 ½ F	UL CRPS	Trauma Significant psychological distress	Chronic pain worsening with movement, allodynia, Hypertrichosis Nail dystrophy Limited ROM	Physical examination *Investigations:* radiography
** Kachko et al., 2008** **Israel**	Retrospective study	*n* = 14 CRPS type 1 and 2 Age: 7–16 (Mage = 11.8) F/M: 10/3	CRPS type 1 (85%) or 2 (15%) 71% female 57% LL CRPS Average time from onset to seeking help was 4.46 weeks (range 0.2–82 weeks). Average referral time to pain clinic was 24.51 weeks (range 1.2–94 weeks)	50% minor trauma 21% surgery 7% severe trauma 7% post infusion 7% arthritis 7% spontaneous	Pain, mechanical hyperalgesia, and allodynia Temperature differences, Skin discolouration Atrophic changes Oedema and hyperhidrosis *Psychosocial Factors:* 32% family or socioeconomic problems	Budapest Criteria *Investigations:* radiographs, isotope bone scan, ultrasound, CT scan, MRI and EMG
** Katholi et al., 2014** **USA**	Review	CRPS type 1 and 2	LLCRPS	Minor trauma	Regional pain disproportionate to trauma, Oedema, Coldness Spasms and tremors Vasomotor and trophic findings	Budapest criteria, Clinical examination *Investigations:* radiography, EMG, sympathetic nerve block, quantitative sensory testing and thermography
** Limerick et al., 2023** **USA**	Systematic literature review	N/A	Mostly females LL CRPS 1 11.5 yrs M age at diagnosis 3–4 ms M duration onset to diagnosis 1.2/1000,000 Estimated incidence	Physical injury; psychological stress; history of atopy	Pain and allodynia Coldness and cyanosis Motor dysfunction with dystonia	Budapest Criteria, clinical examination and diagnosis by exclusion
** Liossi et al., 2015** **UK**	Letter	N/A	Mostly females, LL CRPS	Minor trauma Spontaneous	Pain Severe skin discolouration and temperature changes Mild skin and nail changes Limited ROM and mild tremors	N.R
** Low et al., 2007** **Australia**	Retrospective study	*n* = 20 CRPS Type 1 Age: 8–16 (Mage = 11.8) F/M: 18/2	90% female 85% lower limb 12.1 yrs M onset in F 8.9 yrs M onset in M 13.6 weeks M time to diagnosis (2 days to 41 weeks)	80% minor trauma 20% spontaneous	Continuing pain and allodynia, Oedema, Skin discolouration and temperature changes Trophic changes Limited ROM	Process N.R *Investigations*: radiography, 3-phase technetium bone scan, MRI, and CT scans
**Martínez-Silvestrini & Micheo, 2006** **Puerto Rico**	Case series	*n* = 3 CRPS (Type N.R.) Age: 11–14 F: 3	LL CRPS	Minor trauma	Pain, allodynia Hyperesthesia Swelling Coldness Limited ROM	Physical examination *Investigations:* Imaging studies and radiographs
** Matsui et al., 2000** **Japan**	Case series	*n* = 3 CRPS type 1 and 2 Age: 13–17 F/M: 2/1	66% UL CRPS	Injury; infection	Severe pain, allodynia Swelling Coldness Skin discolouration Cyanosis Hyperhidrosis, Paralysis, stiffness Limited ROM	*Investigations:* Laser doppler flowmetry and sympathetic skin response tests, X ray, MRI
** Murray et al., 2000** **UK**	Retrospective study	*n* = 46 CRPS type 1 Age: 8–15 (Md age = 12.0) F/M: 35/11	76% female with 65% LL CRPS 57% R side affected 39% L side affected 4% R&L affected 6ms M duration onset to diagnosis (range 1–130 weeks)	54% trauma	Severe and continuous pain dysaesthesia, paraesthesia, allodynia, and hyperalgesia Oedema, Mottling Coldness, Altered perspiration, Altered hair growth Limited ROM	Clinical evaluation and history *Investigations:* Radiographs, rheumatological tests, isotope bone scans, ultrasounds, CT scan and MRI
** Okada et al., 1997** **Japan**	Case report	*n* = 1 CRPS type 1 Age: 15 F	UL CRPS	Spontaneous onset	Burning pain, allodynia, dysesthesia and paraesthesia Oedema Coldness Skin discolouration (blue) Limited ROM	*Investigations:* Radiographs, bone scan MRI and rheumatological tests
** Pedemonte Stalla et al., 2015** **Uruguay**	Case series	*n* = 7 CRPS type 1 Age: 7–15 (Mage = 11 yrs) F/M: 6/1	85% female 71% LL CRPS 4–90 days range from symptom onset to diagnosis 56 ± 43 days M Time to diagnosis (range = 4–90 days)	43% trauma 57% spontaneous	Pain, allodynia Coldness, cyanosis, Functional weakness Limb atrophy in late diagnoses *Psychosocial Factors:* Anxiety and depression	Budapest Criteria *Investigations:* radiography, bone scintigraphy study, MRI, EMG, nerve conduction velocity, echo Doppler, immune/inflammatory tests and rheumatoid factor
** Rand, 2009** **USA**	Case report and literature review	*n* = 1 CRPS (type N.R.) Age: 10 F	LL CRPS 7 weeks post injury diagnosis	Ligament tear	Significant pain, allodynia, hyperalgesia Skin discolouration Poor muscle control Non-weightbearing	History, physical examination *Investigations:* Plain radiographs, bone scan, and MRI
** Sethna et al., 2007** **USA**	Prospective study	*n* = 42 CRPS type 1 and 2 Age: 7–17 years (Mage = 13.2) F/M: 40/2	95% female, 100% LL CRPS 12.6ms M symptom duration (range 0.5–72 months)	84% minor trauma 7% fractures 7% post-surgery 2% deep vein thrombosis	Pain, allodynia Skin discolouration and temperature changes Sweating Swelling Altered hair and nail growth Mild muscle atrophy and difficulty weight bearing	IASP Criteria
** Sherry et al., 2023** **USA**	Retrospective study	*n* = 301 CRPS (Type N.R.) Age: 11–15 (Mage = 13 yrs) F/M: 259/42	86% female 79% LL CRPS 93% white ethnic background 7–9 m average pain duration	55% physical trauma 45% spontaneous	Pain and allodynia, Skin discolouration and temperature changes (cold) Swelling Functional neurological disorder symptoms (Stiffness, pseudoseizures, blindness, paralysis, memory loss etc.)	N.R
** Simons et al., 2014** **USA**	Observational fMRI study	*n* = 12 with CRPS (Type N.R.) Age: 10–18 (Mage = 14.1 yrs) F/M: 9/3	75% female, 100% LL CRPS 18.9 ms M pain duration (range = 2.5–85 months)	76% minor trauma 8% fracture 8% surgery 8% spontaneous	91% moderate- severe pain levels 70% reported clinically significant pain-related fear	Budapest Criteria, Neurological examination
** Tan et al., 2008** **Netherlands**	Retrospective study	*n* = 78 CRPS type 1 Age: 5–16 (Md age = 13 yrs) F/M: 67/11	86% female 73% LL CRPS 4.1% UL and LL CRPS	85% minor trauma 15% spontaneous	Pain, hyperesthesia Oedema Abnormal sweating Skin discoloration and temperature changes (cold) Muscle, skin and nail atrophy Tremors, spasms and limited ROM	Veldman et al. 1993 Diagnostic criteria
** Weissmann & Uziel, 2016** **Israel**	Review	N/A	Mostly female Mostly LL CRPS 12 yrs M age at diagnosis CRPS type II can happen younger (3 years onwards)	5–14% Minor trauma; fracture 10–15% surgery psychological factors	Constant pain (described as burning, shooting, stabbing, or electrical) and allodynia Hyperalgesia Swelling Skin discoloration and temperature changes Hyperhidrosis altered hair, skin and nail growth	Physical examination *Investigations:* EMG, plain radiograph, rheumatological tests
** Wilder, 2006** **USA**	Review	N/A	Mostly female, most LL CRPS (5 to 1 ratio) occurring alongside puberty	Trauma; spontaneous	Pain, allodynia disproportionate to inciting event and extended in limb	Physical examination and history *Investigations:* Bone scans
** Youssef et al., 2019** **USA**	Observational fMRI study	*n* = 16 CRPS type 1 Age 10–17 (Mage = 14.3) F/M: 10/6	62% female 100% LL CRPS 3–85 ms M pain duration (15.0 ± 5.4 ms)	N.R	Pain	Budapest Criteria

Abbreviations: *F/M* female/male, *LL* lower limb, *UL* upper limb, *M* mean, *Md* median, *R* right side, *L* left side, *Yrs* years, *Ms* months, *N.R.* not reported, *ROM* range of motion, *EMG* electro myography, *MRI* magnetic resonance imaging, *CT* computed tomography, *pCRPS* Paediatric Complex Regional Pain Syndrome, *IASP* International Association for the Study of Pain

### Treatment

Although the focus of this review was the non-pharmacological management of CRPS in children and young people, a brief summary of medication is given below. Pharmacological treatments summarised here are only for articles that met the inclusion criteria and reported the use of pharmacological techniques as part of a wider treatment programme. For a detailed list of treatment methods see Table 3, where described physiotherapy intervention techniques and types of physical agents used have been detailed as reported. The most common non-pharmacological treatment was physiotherapy (23/30) [6, 10, 19, 21–27, 30, 31, 34–44]. Physiotherapeutic intervention techniques included hydrotherapy, muscle strengthening exercises, desensitisation, active and passive mobilisation and aerobic exercise [6, 10, 19, 21–27, 30–32, 34, 35, 37–44]. Physical agents used included Transcutaneous Electrical Nerve Stimulation (TENS) [6, 10, 13, 32, 43, 44] and Acupuncture [13, 22, 32]. Multidisciplinary treatment was common, and physiotherapy was often delivered alongside medication [6, 10, 19, 21–23, 26, 30–32, 34–36, 38, 40, 42–44], psychological support [6, 10, 13, 19, 21–23, 25–27, 30–32, 36, 39–41, 44], or physical agent interventions [6, 10, 13, 22, 32, 38, 43, 44]. Psychological support was primarily provided through Cognitive Behaviour Therapy (CBT) [10, 13, 21–23, 40, 44]. Guided imagery, visualisation and relaxation techniques were also utilised by few [22, 25, 36]. Pain education was not commonly used [19, 30, 36]. Most studies provided some form of pharmacological treatment [6, 10, 19, 21–23, 26, 30–32, 34–36, 38, 40, 42–44]. Often this included non-steroidal anti-inflammatory drugs (NSAIDs) and analgesics, as well as nerve blockers [6, 10, 19, 21, 22, 26, 30, 32, 34–36, 38, 42–44] or antidepressant medication [19, 21, 32, 38, 40]. Some studies suggested that an interdisciplinary approach to treatment should be prioritised for treatment in this population [13, 19, 21, 25, 26, 34, 40].

### Impact and prognosis

Few studies addressed the impact of pCRPS on patients [6, 10, 19, 22, 23]. Studies that addressed psychosocial impact, suggested dysfunction across a range of parameters [6, 10, 19, 22, 23]. The most reported outcome across studies was absenteeism from school due to pain, and sleep disturbances [10, 19, 23]. A gross decline in physical activity and mood was reported as well [10, 19, 23].

Relapse, reported as a reoccurrence of the condition, was reportedly higher than what is observed in adult populations [10, 13, 23, 31, 32, 40, 41]. Some studies suggested a relapse rate as high as 50% [20, 21]. One review contended that the relapse rates may be gendered, with girls having higher relapse risk and more adverse outcomes in the long run [10]. Many studies had no follow-up periods [28, 30, 33, 35, 38–40, 44]. Studies that did report at follow-up, were often retrospective studies [20, 23, 26, 27, 29, 41], or case reports/series [34, 36, 37, 42, 43] with variable follow-up durations within and across studies (range = 1–34 months). Two prospective studies followed up at a maximum of 4 months [22] and 6 months respectively [6]. Overall, there is some evidence to suggest that prognosis for pCRPS may be positive as some studies suggest a complete return to normal functioning [13, 21, 23, 26, 27, 29, 31, 34, 36, 40–43].

**Table 3:** Treatment and long-term outcomes for pCRPS

**Author/Published year**	**Sample characteristics**	**Interventions**					**Long term outcomes**
		**Physiotherapy/** **rehabilitation approaches**	**Physical agents**	**Psychological**	**Pain education**	**Pharmacological**	**Impact on participants/prognosis**
**Abu-Arafeh & Abu Arafeh, 2016** **UK**	*n* = 26 CRPS type 1 and 2 Age: 5–15 (Mage = 11.9) F/M: 19/7	✓	TENS	✓		✓	88% missed school and stopped sports Families cancelled social activities (73%) and holidays (35%) Parent had to take time off work (35%). Familial stress (35%) parental distress (15%), anxiety (4%), excessive worry (4%) and changing work (4%)
**Abu-Arafeh & Abu Arafeh, 2017** **UK**	N/A						N.R
** Badri et al., 2011 Tunisia **	*n* = 1 CRPS type 1 Age: 13, F	Contrast baths				✓	N.R
** Baerg et al., 2022 Canada **	*n* = 168 CRPS (type N.R.) Age: 2–18 (Mage = 12.2) F/M: 140/28	Fitness, exercise therapy desensitisation		✓	✓	✓	25% missed over 2 weeks of school and 6% enrolled in online school Impacted physical activity (93%), sleep (54%), school achievement (32%), social activities (52%), family function (39%), mood (50%), higher level sport (31%) and withdrawal from treatment (5%)
**Bayle Iniguez et al., 2015** **France**	*n* = 73 CRPS type 1 Age: 5–16 (Mage = 11.5) F/M: 64/9						QOL was significantly lower compared to controls 55% of cases relapsed, 57% reported complete resolution of symptoms, 27% improved and 16% had no change at 12 months follow up
** Borucki & Greco, 2015** **USA**	N/A	✓		✓		✓	pCRPS patients have a milder disease course than adults Cure rates range between 46 and 92%. Recurrence rates are high (20–50%)
** Broman et al., 2021** **Germany**	*n* = 28 health care professionals	Mirror therapy, exercise, music therapy, and Scottish baths	TENS	✓		✓	N.R
** Brooke & Janselewitz, 2012** **USA**	*n* = 32 CRPS type 1 Age: 8–18 (Mage = 14.3) F/M: 26/6	high intensity aerobic exercises and occupational therapy, desensitization, fluidotherapy 5 h per day, 5 days a week		✓			34% had complete resolution at discharge and 44% had complete resolution within 2 months Of 19 participants at follow-up, 9% had incomplete pain resolution, but improved 22% relapsed, 86% achieved full resolution
** Chalkiadis, 2001** **Australia**	*n* = 44 CRPS type 1 and 2 Age: 9–17 (Mage = 13.7) F/M: 33/11	✓	Acupuncture	✓		✓	77% had marked reduction in pain and marked functional improvement 16% had some reduction in pain and some functional improvement 7% had no reduction in pain and no functional improvement 66% missed over 40 days of school
** di Vadi et al., 2002** **UK**	*n* = 2 CRPS type 1 Age: 10 and 13 F: 2	✓				✓	Good outcomes at 1 month post treatment
** Dunbar & Wilson, 2019** **New Zealand**	n = 1 CRPS type 1 Age: 15 F			✓	✓	✓	Rapid progress and reduction/loss of CRPS symptoms was noted at 16 days and at 5 weeks post initial assessment
**Güler-Uysal & Bas, 2003** **Turkey**	*n* = 1 CRPS type 1 Age: 2 ½ F	✓ occupational therapy				✓	Good recovery was reported at 6 and 12 month follow up
** Kachko et al., 2008** **Israel**	*n* = 14 Suspected CRPS type 1 and 2 Age: 7–16 (Mage = 11.8) F/M: 10/3	✓		✓		✓	Full recovery in 78.5% Most had reduced pain and improved function. Average time of treatment was 8 weeks (range 2–28 weeks) School absences of more than twice a week in 47%
** Katholi et al., 2014** **USA**	CRPS type 1 and 2	✓ aquatic walking exercises, contrast baths, fluidotherapy, and elastic therapeutic tape. Progressive pressure, tactile desensitization, hot/cold packs	Acupuncture, TENS	✓			Prognosis is good however recurrence rates are high (30%)
** Limerick et al., 2023** **USA**	N/A	✓ rehabilitation and hydrotherapy, desensitisation					N.R
** Liossi et al., 2015** **UK**	N/A	Active remobilisation, desensitisation, muscle strengthening and restoring normal function		✓			Condition severely affected psychological functioning Functional outcomes were good; recurrence rates were high
** Low et al., 2007** **Australia**	*n* = 20 CRPS Type 1 Age: 8–16 (Mage = 11.8) F/M:18/2	✓		✓		✓	20% relapsed, 5% relapsed twice 70% had only 1 episode of CRPS with complete resolution of symptoms. 10% were admitted to a hospital. Mean time to symptom resolution = 17.6 weeks
**Martínez-Silvestrini et al., 2006** **Puerto Rico**	*n* = 3 CRPS (Type N.R.) Age: 11–14 F: 3	✓ increasing weight bearing capacity, hydrotherapy, and desensitization	High voltage electrical stimulation			✓	Relapse observed in one patient
** Matsui et al., 2000** **Japan**	*n* = 3 CRPS type 1 and 2 Age: 13–17 F/M: 2/1	✓		✓			N.R
** Murray et al., 2000** UK*	*n* = 46 CRPS type 1 Age: 8–15 (Md age = 12.0) F/M: 35/11	✓		✓			7% continued to have symptoms 24 months from diagnosis. Median recovery time was seven weeks from diagnosis (range = 1 to 140 weeks) 24% relapsed at follow up
** Okada et al., 1997** **Japan**	*n* = 1 CRPS type 1 Age: 15 F		TENS			✓	Good recovery with return to normal activity over 6 months
**Pedemonte-Stalla et al., 2015** **Uruguay**	*n* = 7 CRPS type 1 Age: 7–15 (Mage = 11 yrs) F/M: 6/1	✓ Occupational therapy Hydrotherapy and cutaneous desensitisation				✓	Response was excellent and no relapses were noted
** Rand, 2009** **USA**	*n* = 1 CRPS (type N.R.) Age: 10 F	✓		✓		✓	Prognosis is variable, recurrence rate was high, physical therapy was beneficial
** Sethna et al., 2007** **USA**	*n* = 42 CRPS type 1 and 2 Age: 7–17 yrs (Mage = 13.2) F/M: 40/2						N.R
** Sherry et al., 2023** **USA**	*n* = 301 CRPS (Type N.R.) Age: 11–15 (Mage = 13 yrs) F/M: 259/42						48% were fully functional, remaining continued to report symptoms (pain) Over 50% had no subsequent pain and over 70% had no other clinical symptoms
** Simons et al., 2014** **USA**	*n* = 12 with CRPS (Type N.R.) Age: 10–18 (Mage = 14.1 yrs) F/M: 9/3	✓ Occupational therapy		✓	✓		N.R
** Tan et al., 2008** **Netherlands**	*n* = 78 CRPS type 1 Age: 5–16 (Md age = 13 yrs) F/M: 67/11	✓		✓		✓	28% relapsed. 19% due to new injury. 6% had two relapses, 1% with three and 3% with four relapses 60% relapses were in the same limb with similar presentation
** Weissmann & Uziel, 2016** **Israel**	N/A	✓ Mobilisation and massage	TENS	✓		✓	School is more stressful, school related problems and absences are common Recurrence rates are high, and pain lasts much later in life with men faring better than women over time
** Wilder, 2006** **USA**	N/A	✓	TENS, Acupuncture	✓		✓	Recurrence rates are high
** Youssef et al., 2019** **USA**	*n* = 16 CRPS type 1 Age 10–17 (Mage = 14.3) F/M: 10/6						N.R

Key *F/M* female/male, *M* mean, *Yrs* year, *N.R.* not reported, *TENS* transcutaneous electrical nerve stimulation, *QOL* quality of life; blank cells: treatment not described

## Discussion

This scoping review aimed to answer the question “How is CRPS characterised, diagnosed and treated in under 18-year-olds?” by (1) describing the characteristics, (2) reporting diagnostic approaches, and (3) presenting the range of treatments. We present our findings aligned to these objectives in the context of the current literature and conclude by summarising the gaps in knowledge and identifying key areas for research and practice.

### Clinical characteristics

Consistent with the adult clinical diagnostic (Budapest) criteria [45], spontaneous and allodynic pain were the primary symptoms alongside vasomotor, sudomotor, and trophic changes. Higher rates of movement disorders were reported in pCRPS (30%) [14] compared to adults (25%) [46]. Additionally, paralysis, seizures, blindness and other functional neurological symptoms not included in the adult diagnostic criteria were reported [29, 39]. Insufficient detail was provided to ascertain exactly which symptoms of functional neurological disorder were outside of the Budapest motor dysfunction criteria (comprising reduced range of movement, weakness, tremor or dystonia). This clinical overlap between functional neurological disorder and CRPS in under 18-year-olds may be due to potentially similar pathologies in malfunctioning nervous systems [8].

### Diagnosis

Only a third of studies used the Budapest criteria, despite being the recognised diagnostic criteria for CRPS, albeit for adults [45]. Six records (20%) failed to report how their study populations were diagnosed as such it is difficult to say how accurate their diagnosis of pCRPS is likely to be. Others used outdated adult diagnostic criteria such as Veldman et al. (1993) and Stanton et al. (1993), despite being superseded by the Budapest Criteria at the time of publication. Although no recognised diagnostic biomarkers exist, clinical investigations, typically blood tests and scans such as X-rays, ultrasound, bone scans, CT, and MRIs were common either alongside a physical examination or as standalone means of diagnosis [9].

The reported incidence of pCRPS, 1.14–1.16/100 000 person years [6, 19] is considerably lower than that in adults (estimates up to 26.2 per 100,000 person years) [5]. Paediatric estimates are less accurate as based on smaller population sizes (reported incidence from 600 clinicians [6] and 2,268 [19] paediatricians) compared to adults (600,000 patients) [5]. A twenty-fold variation between the two estimates could be, in part, associated with an overestimation in adults due to the use of outdated international classification disease codes [9]. However, a significant explanatory factor is the underdiagnosis of CRPS in children. Without validated diagnostic criteria in children, pCRPS is harder to identify and treat effectively. This may contribute to confusion amongst clinicians about what constitutes pCRPS, leading to misdiagnosis and attributing symptoms of limb pain and swelling to other causes, such as infection or ongoing tissue trauma. Evidence suggests that children may describe and show pain differently from adults, as such using age and developmentally appropriate tools to capture pain in younger populations may support more accurate diagnoses [47].

### Treatments

Physiotherapy was the primary non-pharmacological treatment for pCRPS. However, a recent Cochrane review suggested limited evidence for physiotherapy in improving adult CRPS [48]. A range of non-pharmacological treatments have been used with varying effectiveness among adults [48, 49]. Interestingly, only some of these approaches were used with paediatric populations. In a recent review, Griffiths and colleagues found extensive usage of sensory retraining as a form of non-pharmacological treatment among adults [49]. Contrastingly, no studies for pCRPS used sensory retraining as a treatment approach. Despite the differences in treatment modalities for adult and paediatric CRPS, Griffiths and colleagues echo the findings of this scoping review in recommending an interdisciplinary approach to CRPS management [49]. It is also worth noting that despite limited benefits of non-steroidal anti-inflammatory drugs among adults, they were used ubiquitously in pCRPS management [50, 51]. The same was true for anticonvulsants and antidepressants [51]. Furthermore, where antidepressants were reported, it was unclear whether they were prescribed to treat pCRPS pain or to manage the psychological impact of the condition. The absence of a unified approach to pCRPS management was evident across studies. The heterogeneity of treatment and the usage of treatments with limited or no evidence of efficacy highlight the need for clinical trial evidence and consensus-based guidelines to improve pCRPS management.

### Impact

This review highlights a gap in the psychosocial management of pCRPS. Although evidence suggests a strong preponderance of disease incidence around puberty, few studies document the psychological impact of pCRPS and fewer cater to its management. Prolonged periods of school absences, as observed with pCRPS can impact relationships, academics and identity development [52]. Absenteeism has been associated with lower social engagement, and an added risk of loneliness and isolation in young people with chronic pain [52]. It can impact health-related quality of life and peer relationships, impacting psychological well-being [52, 53]. In a study looking at distress in children with CRPS, Logan and colleagues found greater functional impairment and physical disability among children with CRPS compared to other pain diagnoses [53]. Further, studies addressing other chronic pain conditions among adolescents suggest an overall lower quality of life, fewer peer relationships and higher peer victimisation as compared to their pain-free counterparts [54]. Managing the psychosocial distress and functional impairments that accompany pCRPS needs to be prioritised in the development of future treatment pathways.

### Gaps in knowledge and future directions

Following the synthesis of results, the PAGER framework was used to summarise and identify key areas for research and practice. Three broad categories of *identification*, *treatment* and *impact* were generated from the synthesised data via the PAGER Framework (see Table 4). *Identification* revealed the lack of agreed pCRPS diagnostic criteria as a primary point of concern. pCRPS *treatment* highlighted the absence of agreed clinical treatment guidelines. Finally, *impact* exposed the limited understanding in living with pCRPS, from the child’s perspective and that of their family. The identified key recommendations are:

- Develop international consensus-driven diagnostic criteria for pCRPS using evidence-based strategies such as diagnostic studies.
- Conduct large pCRPS population studies to accurately estimate incidence and prevalence.
- Develop age and developmentally appropriate treatment guidelines.
- Understand the child’s experience of living with pCRPS, the impact on their lives and that of their family and wider community.

Despite the dearth of research in this field, the need to prioritise a patient-centred approach in research and practice is evident. Additionally, ensuring age-appropriate needs are explored in research will improve patient outcomes in practice.

**Table 4:** PAGER framework summarising priority areas for research and practice

Pattern	Advances	Gaps	Evidence for practice	Research recommendations
*Identification*	Growing awareness and recognition of CRPS and the range of clinical presentations in children and young people	No agreed pCRPS diagnostic criteria	Clinical presentation comprises pain and a range of other symptoms Clinical examination and the Budapest Criteria (not validated in CYP) is presently considered most reliable Clinical investigations do not determine diagnosis	Conduct primary diagnostic studies to more accurately document clinical features of pCRPS Develop internationally recognised, consensus driven diagnostic criteria for pCRPS Conduct large pCRPS population studies to accurately estimate incidence and prevalence
*Treatment*	Recognising the need for a multidisciplinary treatment approach lack of evidence-based treatment	No agreed clinical treatment guidelines	Rehabilitation has consistently shown good results especially alongside psychological support Medication shows little to no efficacy	Develop evidence-based consensus treatment guidelines for pCRPS that align with age and developmental stages Conduct appropriately designed clinical trials to understand treatment efficacy
*Impact*	Some awareness of the impact on quality of life, schooling and family	Limited understanding of the child’s experience and impact of CRPS on their lives	Prognosis of the condition is good; however relapse rates are high	Improve understanding of the child’s experience and impact on their life/developmental stages/psychosocial functioning and wider impact on parents/society and transition to adulthood

Abbreviations:* CYP *Children and Young People.

### Strengths and limitations

When considering strenghts, this review highlights prominent symptoms, diagnostic tools and treatment approaches for pCRPS. By utilising the PRISMA format for reporting scoping reviews and registering on the open science framework, it ensures rigour and transparency of the review findings. Additionally, highlighting prominent gaps in research and practice emerging from the PAGER framework, provide clear directions for researchers and health professionals. Finally, the broad scope of review meant the inclusion of a range of study methodologies due to which a quality appraisal was not possible [18]. Locating potential studies via expressions of interest was not conducted during the study search for this review. Additionally, it is important to note that the pharmacological management for pCRPS noted in this review only pertains to articles that met the criteria for non-pharmacological management. Often in these studies, pharmacological management was reported as one part of a wider multidisciplinary treatment approach. Therefore, while we have summarised findings for pharmacological methods used as well as the impact and prognosis of pCRPS, this was not the primary aim of the review. As such findings regarding these factors may not be exhaustive. Further, Randomised Control Trials were considered outside the scope of this review as our goal was to seek detailed descriptions of the presentation, diagnosis and treatment for pCRPS, but recognise that their exclusion is a limitation. Another limitation was the exclusion due to language as most included studies were from UK and USA. Few studies were from the Middle East (Israel, 2/30; [10, 23] Turkey 1/30) [37], Asia (Japan, 2/30) [39, 43], South America (Uruguay, 1/30) [34], or Africa (Tunisia, 1/30) [35]. Although some research suggests this does not have a large bearing on findings [55], it is important to understand how pCRPS presents in regions other than the west in future research.

## Conclusion

This review presents the limited evidence base for pCRPS and provides a clear direction to better understand and treat pCRPS. It highlights the importance of specific diagnostic criteria for paediatric populations and the need for consensus driven treatment guidelines. Further research involving children and young people is needed to elucidate the experience and impact of pCRPS, specifically qualitative explorations, diagnostic studies and studies to determine treatment efficacy. This will improve understanding and future management of this painful condition.

## Supplementary Information

Below is the link to the electronic supplementary material.

ESM 1 (DOCX 109 KB)

ESM 2 (DOCX 23.1 KB)

## Data Availability

No datasets were generated or analysed during the current study.
