# Supplementary material for: Characterisation and non-pharmacological treatment of Complex Regional Pain Syndrome in under-18-year-olds: a scoping review
Source: Eur J Pediatr. 2026 May 8;185(6):364. doi: 10.1007/s00431-026-07027-w (PMC13152901; doi:10.1007/s00431-026-07027-w)
Supplement: Supplementary file 2 — (DOCX 23.1 KB) [file 431_2026_7027_MOESM2_ESM.docx]

**Supplementary Materials:** *Characterisation and non-pharmacological treatment of complex regional pain syndrome in under 18-year-olds: A scoping review*

*Authors: Mahira Budhraja, Ryan Purvis, Katrina Tang and Dr. Jennifer Lewis.*

**Table 1.** *Search Strategy*

| **Search Number** | **Search terms** |
| --- | --- |
| Search 1 | - “Non-adults” or “under-18”, or “under eighteen”, or juvenile, or kid* or minor or youngster or “PEADs” or “paediatric” or youth*. |
| Search 2 | - “CRPS,” or “complex regional pain syndrome.” |
| Search 3 | - Acute hospital* or acute care hospitals or Hospitals, Clinic* or Physiotherapy or “community hospitals” or private hospitals or school* |
| Search 4 | - Search 1 AND search 2 |
| Search 5 | - Search 3 AND search 4 |

**Table 2**. *Summary characteristics of included studies.*

| **Study Characteristics**  **N** | |
| --- | --- |
| ***Study type*** |  |
| Retrospective studies | 7 |
| Case Reports and Series | 9 |
| Reviews | 6 |
| Prospective studies | 3 |
| Observational fMRI Studies | 2 |
| Surveillance studies | 2 |
| Letters to the editor | 1 |
| ***Country*** |  |
| USA | 10 |
| UK | 5 |
| Australia | 2 |
| Israel | 2 |
| Japan | 2 |
| Canada | 1 |
| France | 1 |
| Germany | 1 |
| Netherlands | 1 |
| New Zealand | 1 |
| Puerto Rico | 1 |
| Turkey | 1 |
| Tunisia | 1 |
| Uruguay | 1 |
| ***Location of CRPS (identified or described)*** | |
| Majority lower limb | 20 |
| Upper limb only | 4 |
| Lower limb only | 4 |
| Majority upper limb | 1 |
| Not Described | 1 |
